# The effect of cardiolipin side chain composition on cytochrome c protein conformation and peroxidase activity

**DOI:** 10.14814/phy2.14772

**Published:** 2021-03-05

**Authors:** Jennifer A. Wilkinson, Sebastian Silvera, Paul J. LeBlanc

**Affiliations:** ^1^ Center for Bone and Muscle Health Faculty of Applied Health Sciences Brock University 1812 Sir Isaac Brock Way St. Catharines Ontario L2S 3A1 Canada

**Keywords:** apoptosis, cytochrome c peroxidase, muscular dystrophy, synthetic membranes

## Abstract

Skeletal muscle, a highly active tissue, makes up 40% of the total body weight. This tissue relies on mitochondria for ATP production, calcium homeostasis, and programed cell death. Mitochondrial phospholipid composition, namely, cardiolipin (CL), influences the functional efficiency of mitochondrial proteins, specifically cytochrome c. The interaction of CL with cytochrome c in the presence of free radicals induces structural and functional changes promoting peroxidase activity and cytochrome c release, a key event in the initiation of apoptosis. The CL acyl chain degree of saturation has been implicated in the cytochrome c to cytochrome c peroxidase transition in liposomal models. However, mitochondrial membranes are composed of differing CL acyl chain composition. Currently, it is unclear how differing CL acyl chain composition utilizing liposomes will influence the cytochrome c form and function as a peroxidase. Thus, this study examined the role of CL acyl chain saturation within liposomes broadly reflecting the relative CL composition of mitochondrial membranes from healthy and dystrophic mouse muscle on cytochrome c conformation and function. Despite no differences in protein conformation or function between healthy and dystrophic liposomes, cytochrome c's affinity to CL increased with greater unsaturation. These findings suggest that increasing CL acyl chain saturation, as implicated in muscle wasting diseases, may not influence cytochrome c transformation and function as a peroxidase but may alter its interaction with CL, potentially impacting further downstream effects.

## Graphical abstract

Cardiolipin fatty acyl composition is typically polyunsaturated and is important for the interaction with cytochrome c and its dual role as both an electron shuttle and peroxidase. This study's findings suggest that increasing cardiolipin acyl chain saturation, as seen in muscle wasting diseases, may not influence cytochrome c transformation and function as a peroxidase but may alter its interaction with cardiolipin, potentially impacting further downstream effects, such as apoptosis.

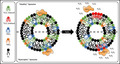

## 1 INTRODUCTION

Cytochrome c is a small heme protein loosely associated with the inner membrane space (IMS)‐facing leaflet of the inner mitochondrial membrane (IMM) (Alvarez‐Paggi et al., 2017). This protein is best known as an electron shuttle between complexes III and IV in the electron transport chain during oxidative phosphorylation (Alvarez‐Paggi et al., 2017), but also plays a critical proapoptotic role. The release of cytochrome c is preceded by a series of orchestrated events that is dependent on its interaction with cardiolipin (CL), a mitochondrial specific phospholipid.

Cardiolipin is an anionic phospholipid predominantly located on the matrix‐facing leaflet of the IMM (Daum, 1985; Gonzalvez & Gottlieb, 2007; Horvath & Daum, 2013). Small amounts of CL found on the IMS‐facing leaflet of the IMM interact with cytochrome c through two proposed mechanisms. Loosely bound electrostatic interactions allows cytochrome c to function as an electron shuttle, whereas tighter hydrophobic interactions transform cytochrome c into a peroxidase (Gonzalvez & Gottlieb, 2007; Huttemann et al., 2011; Kagan et al., 2005; Mohammadyani et al., 2018). The hydrophobic interaction involves the protrusion of a CL acyl chain into the hydrophobic cleft of cytochrome c (Alvarez‐Paggi et al., 2017). This hydrophobic interaction‐mediated transformation of cytochrome c into a peroxidase has been identified as a critical first step in the apoptotic pathway (Kagan et al., 2005). However, previous studies have demonstrated that this transformation appears to be dependent on the degree of saturation of the CL acyl chains (Abe et al., 2011; Belikova et al., 2006). This acyl chain‐specific transformation of cytochrome c to a peroxidase may have implications in skeletal muscle diseases that have altered mitochondrial membrane lipid composition.

Previous research determining the effect of CL acyl chain composition have used single fatty acyl species using liposome models. However, CL in mitochondrial membranes is composed of differing acyl chain compositions. In healthy skeletal muscle mitochondria, 40–70% of CL is comprised of 18:2n6, depending on the muscle examined, with the remaining 30–60% composed of mainly 16:0, 18:0, and 18:1 (Stefanyk et al., 2010; Tsalouhidou et al., 2006). Under pathological conditions such as Duchenne muscular dystrophy (DMD), human and preclinical rodent model skeletal muscle phospholipid acyl chain composition shifts from 18:2n6 to 18:1 (Benabdellah et al., 2009; Tahallah et al., 2008; Touboul et al., 2004), which holds true when examining CL (Zibamanzarmofrad, 2015; Zibamanzarmofrad et al., 2013). It is currently unknown if cytochrome c's form and function changes with liposomes that broadly reflect the percent CL composition of mitochondrial membranes similar to liposomes that contain one species of CL. In addition, it is currently unknown if the shift in CL acyl chain composition seen under dystrophic conditions will translate into altered CL‐cytochrome c interaction. Therefore, the current study examined the influence of CL acyl chain composition broadly reflecting the percent CL composition of healthy and dystrophic muscle mitochondrial membranes on cytochrome c conformation, binding affinity, and peroxidase activity.

## 2 MATERIALS AND METHODS

### 2.1 Materials

1’,3’‐bis[1,2‐dimyristoyl‐sn‐glycero‐3‐phospho]‐sn‐glycerol (tetramyristoyl cardiolipin, TMCL, 14:0) and 1’,3’‐bis[1,2‐dioleoyl‐sn‐glycero‐3‐phospho]‐sn‐glycerol (tetraoleoyl cardiolipin, TOCL, 18:1) were purchased from Avanti Polar Lipids (Alabaster, Alabama, USA). Bovine heart cardiolipin (BHCL; ≥80% tetralinoleoyl cardiolipin, TLCL, 18:2n6), L‐α‐phosphatidylcholine (PC), and cytochrome c (equine heart) were purchased from Sigma‐Aldrich Canada. 10‐acetyl‐3,7‐dihydroxyphenoxazine (ADHP) was purchased from Cayman Chemicals.

### 2.2 Preparation of liposomes and determination of size

Liposomes were created by mixing equal ratios of PC and CL as per Birk et al. (Birk et al., 2015). The cardiolipin component consisted of either one type of cardiolipin species (TMCL, TOCL, BHCL) or a mixture of cardiolipin species that broadly reflected the CL percent composition of healthy (Con; 39% TLCL, 6% TOCL, 5% TMCL) or dystrophic (Dys; 28% TLCL, 17% TOCL, 5% TMCL) skeletal muscle mitochondria (Zibamanzarmofrad et al., 2013). Briefly, lipids were mixed into a small glass vial and dried under a steady stream of nitrogen gas and resuspended in 32 mM HEPES buffer. Each sample was then placed on ice and sonicated (2 times 30 s with a probe intensity of 2) using a tip sonicator (Sonic Dismembrator Model 100, Fisher Scientific, Ottawa, Ontario, Canada). Liposome samples (50 µM) were assessed for size using a Protein Solutions DynaPro‐99‐E‐50 Dynamic Light Scattering Module (Santa Barbara, California, USA).

### 2.3 Cytochrome c peroxidase activity

The cytochrome c peroxidase activity was assessed by measuring the conversion of ADHP to the fluorescent by‐product resorufin as previously reported (Birk et al., ,^2013^, ^2015^; Mandal et al., 2015). Briefly, cytochrome c (2 µM) was incubated with the previously mentioned liposomes (30 µM) and peroxidase activity was initiated by the addition of 50 µM H_2_O_2_, which is similar to the work of Mandal et al (Mandal et al., 2015) and in the range of H_2_O_2_ (10–100 µM) used in the previous literature examining cytochrome c peroxidase activity (Abe et al., 2011; Belikova et al., 2006; Birk et al., ,^2013^, ^2015^; Kapralov et al., 2011). The cytochrome c peroxidase activity was monitored fluorometrically (excitation at 530 nm and emission at 590 nm) using a plate reader (Synergy HT‐1 Plate Reader, Bio‐Tek, Winooski, Vermont, USA).

### 2.4 Ultraviolet‐visible absorption spectrophotometry for cardiolipin oxidation

Two lots of commercially available BHCL samples suspended in chloroform:methanol were purchased from Sigma approximately a year apart and remained stored at −20^0^C. Differences in the cytochrome c peroxidase activity between BHCL lots raised concerns about possible peroxidation. To test for oxidation, both BHCL lots were suspended in HEPES buffer in a quartz cuvette to a final concentration of 100 µM. The absorbance at 234 nm was measured (Ultraspec 2100 Pro, UV/Visible spectrophotometer, Biochrom, Massachusetts, USA) to assess the formation of conjugated dienes as an indirect measure of polyunsaturated lipid peroxidation (Lokhmatikov et al., 2014).

### 2.5 Assessment of cytochrome c structural properties

Samples containing the various PC:CL liposomes (500 µM) and cytochrome c (50 µM) were incubated in quartz cuvettes for 15 minutes at room temperature and away from light to induce cardiolipin‐cytochrome c complex formation (Birk et al., 2013). Absorption across the 200–900 nm spectrum was measured (Ultraspec 2100 Pro, UV/Visible spectrophotometer, Biochrom, Massachusetts, USA) to determine the influence of cardiolipin on cytochrome c protein unfolding, with a focus on the peak absorbance and wavelength of the Soret and Q bands (Giovannetti, 2012).

### 2.6 Cardiolipin‐cytochrome c binding strength

Measurements of cardiolipin‐cytochrome c binding were previously reported (Belikova et al., 2006) with the following changes. Samples of 20 µM cytochrome c in HEPES buffer (pH 7.4) were incubated with or without 1 mM of respective CL:PC liposomes for 30 minutes with intermittent shaking at room temperature. Samples were then incubated with varying concentrations of nonyl‐acridine orange (0–9 mM) for one hour at room temperature, as per Petit et al. (Petit et al., 1992). Samples were filled with equivalent volume of sample buffer (25% v/v, 62.5 mM Tris HCl, pH 6.8), loaded onto an 8% native polyacrylamide gel and run for one hour at 150 V. Gels were removed and underwent a series of incubations with constant rocking at room temperature; one hour with 0.2% Coomassie blue R‐250, two hours with destaining solution (20% ethanol v/v, 5% acetic acid v/v, 1% glycerol v/v, in distilled water), overnight in distilled water, and finally one hour in the destaining solution. Gels were imaged using iScanner Pro and analyzed with Image Studio (Li‐Cor, Lincoln, Nebraska, USA). The density of bands with cytochrome c and CL:PC liposomes was compared to cytochrome c only, and the binding constant (*K_b_*) was calculated as per previously reported (Belikova et al., 2006; Petit et al., 1992).

### 2.7 Statistics

All values are expressed as the mean ± standard error (SE). All statistical analyses were performed using SPSS Statistics for Windows, version 25 (SPSS Inc., Chicago, Ill., USA). One‐way ANOVAs were performed on all data sets followed by a Tukey's post hoc analysis. Assumption for normality was verified by generating appropriate residual plots. Regression analysis was conducted between Soret and Q band peak absorbance and cytochrome c peroxidase activity. A *p* value of <0.05 was considered significant for all tests.

## 3 RESULTS

### 3.1 Liposome size

**FIGURE 1:**
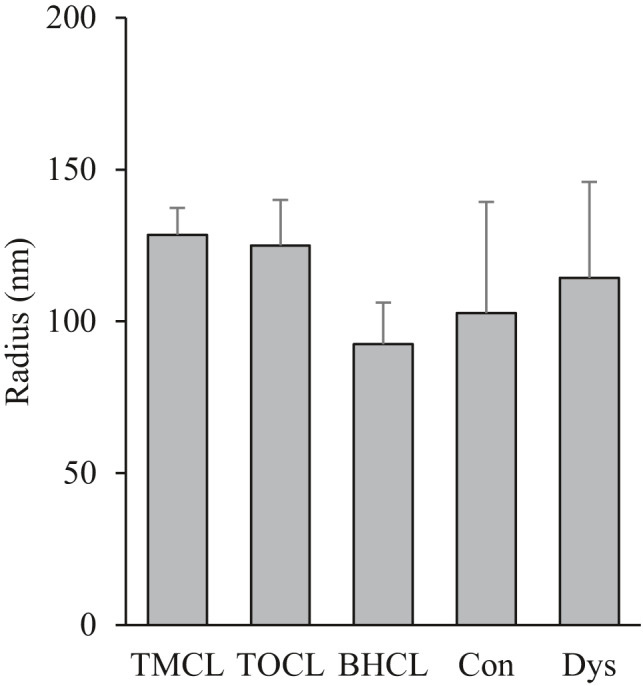
Liposome size measured immediately after sonication. Values are mean ±SEM, n = 3 for each condition. TMCL, 1’,3’‐bis[1,2‐dimyristoyl‐sn‐glycero‐3‐phospho]‐sn‐glycerol cardiolipin; TOCL, 1’,3’‐bis[1,2‐dioleoyl‐sn‐glycero‐3‐phospho]‐sn‐glycerol cardiolipin; BHCL, bovine heart cardiolipin; Con, liposome broadly reflecting the percent cardiolipin composition of normal skeletal muscle mitochondria; and Dys, liposome broadly reflecting the percent cardiolipin composition of dystrophic skeletal muscle mitochondria.

The liposome size was not different between liposomes of differing CL composition (Figure 1), thus confirming that other comparatives between CL species were not an artifact of differing liposome sizes.

### 3.2 Cytochrome c peroxidase activity is enhanced with unsaturated cardiolipin side chains

**FIGURE 2:**
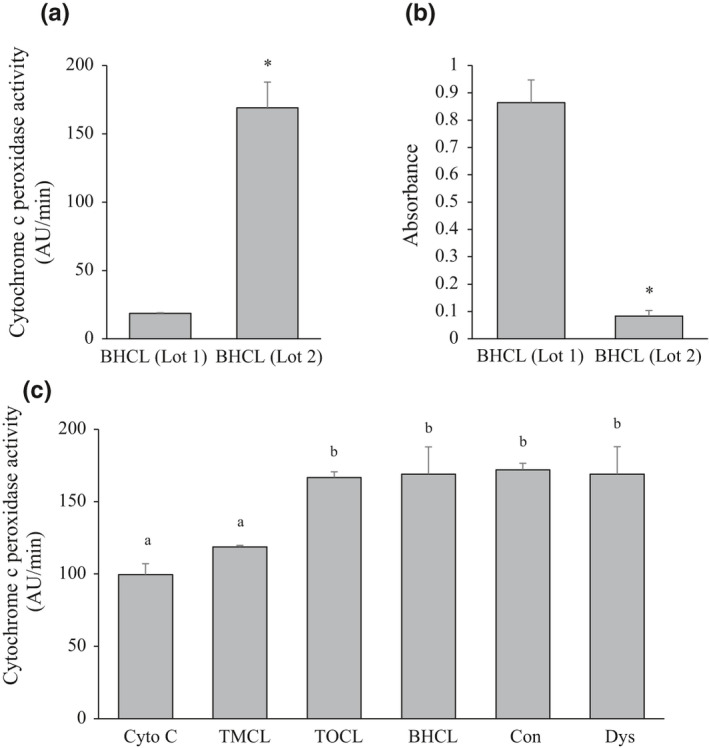
Cytochrome c peroxidase activity is enhanced in the presence of cardiolipin with non‐oxidized unsaturated acyl side chains at 50 µM H_2_O_2_. (a) Cytochrome c peroxidase activity with bovine heart cardiolipin from two separate lots at 50 µM H_2_O_2_. (b) Spectrophotometric assessment of conjugated dienes within the side chains of polyunsaturated cardiolipin species without cytochrome c from two separate lots as a surrogate measure of cardiolipin peroxidation. (c) Cytochrome c peroxidase activity with and without cardiolipin of varying species at 50 µM H_2_O_2_. Values are mean ±SEM, n = 3 for each condition; * indicates the significant difference from BHCL (Lot 1); no statistical difference between values with the same letter (*p* < 0.05). AU, arbitrary units; Cyto C, cytochrome c; TMCL, 1’,3’‐bis[1,2‐dimyristoyl‐sn‐glycero‐3‐phospho]‐sn‐glycerol cardiolipin; TOCL, 1’,3’‐bis[1,2‐dioleoyl‐sn‐glycero‐3‐phospho]‐sn‐glycerol cardiolipin; BHCL, bovine heart cardiolipin; Con, liposome broadly reflecting the percent cardiolipin composition of normal skeletal muscle mitochondria; and Dys, liposome broadly reflecting the percent cardiolipin composition of dystrophic skeletal muscle mitochondria.

The cytochrome c peroxidase activity at 50 µM H_2_O_2_ with BHCL Lot 1 liposomes (Figure 2a) was identified to be significantly lower than that reported in the literature (Abe et al., 2011). A second lot of BHCL (Lot 2) resulted in a significantly higher cytochrome c peroxidase activity. As the lot integrity was in question, diene conjugation, a surrogate of polyunsaturated lipid peroxidation, was measured (Figure 2b). As a result, BHCL Lot 1 had significantly higher absorbance at 234 nm, indicating greater polyunsaturated lipid peroxidation compared to BHCL Lot 2, and all subsequent data were collected using BHCL Lot 2.

Compared to cytochrome c alone, the cytochrome c peroxidase activity at 50 µM H_2_O_2_ increased ~50% with monounsaturated (TOCL), polyunsaturated (BHCL), and both mixed CL liposomes broadly reflecting control (Con) and dystrophic (Dys) mitochondrial cardiolipin percent composition (Figure 2c). By contrast, saturated (TMCL) cardiolipin species resulted in a small (19%) but not significant (*p* = 0.07) increase in peroxidase activity compared to cytochrome c alone.

### 3.3 Incubating cytochrome c with cardiolipin species influences the folded and unfolded states of cytochrome c

**FIGURE 3:**
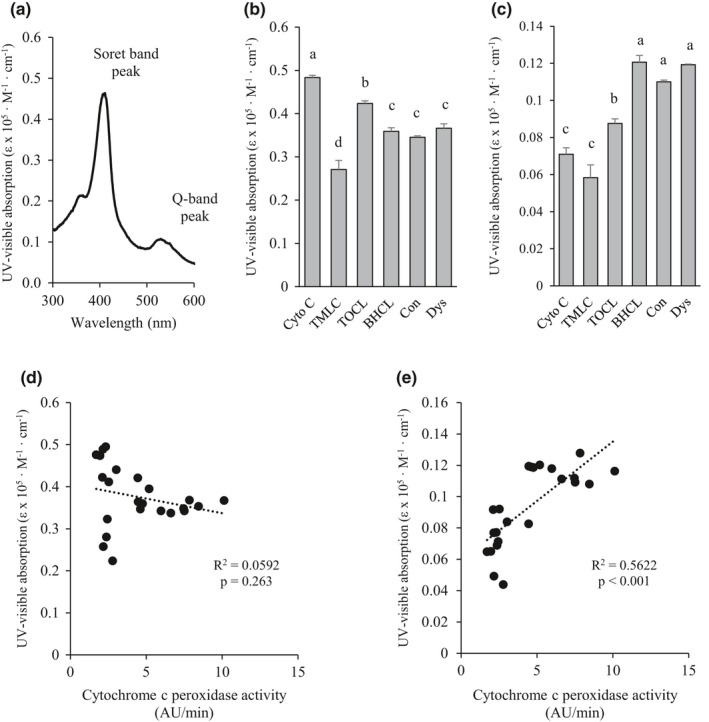
Incubation of cytochrome c with cardiolipin species containing different acyl side chains influences the folded and unfolded states of cytochrome c, but these changes do not correlate with cytochrome c peroxidase activity. (a) Representative ultraviolet‐visible spectrum demonstrating the Soret and Q band peaks; maximal absorbance of the (b) Soret and (c) Q bands of cytochrome c with or without cardiolipin of varying acyl side chain composition; correlation between cytochrome c peroxidase activity using ADHP and maximal (d) Soret and (e) Q band absorbance. Values are mean ±SEM, n = 4 for each condition; no statistical difference between values with the same letter (*p* < 0.05). Cyto C, cytochrome c; TMCL, 1’,3’‐bis[1,2‐dimyristoyl‐sn‐glycero‐3‐phospho]‐sn‐glycerol cardiolipin; TOCL, 1’,3’‐bis[1,2‐dioleoyl‐sn‐glycero‐3‐phospho]‐sn‐glycerol cardiolipin; BHCL, bovine heart cardiolipin; Con, liposome to broadly reflecting the percent cardiolipin composition of normal skeletal muscle mitochondria; and Dys, liposome to broadly reflecting the percent cardiolipin composition of dystrophic skeletal muscle mitochondria.

The absorption spectrum of cytochrome c alone demonstrated a Soret band peak at 409 nm and a Q band peak at 528 nm (Figure 3a). In comparison to cytochrome c alone, the peak wavelength did not differ for the Soret or Q band for each liposome (data not shown). However, compared to cytochrome c alone, the peak absorbance decreased for the Soret band (cyto C > TOCL >BHCL = Con =Dys > TMCL) (Figure 3b) and increased for the Q band (cyto c = TMCL <TOCL < BHCL =Con = Dys) (Figure 3c). The Soret and Q bands are quantitative regions of the UV–Vis spectra that identify cytochrome c conformational changes (Nantes et al., 2001), which in turn may influence peroxidase activity. As such, regression analysis demonstrated that increased Q band peak absorbance increased the cytochrome c peroxidase activity (Figure 3e), which was not seen with the Soret band (Figure 3d).

### 3.4 Binding affinity of cytochrome c is influenced by the presence of polyunsaturated cardiolipin species

**FIGURE 4:**
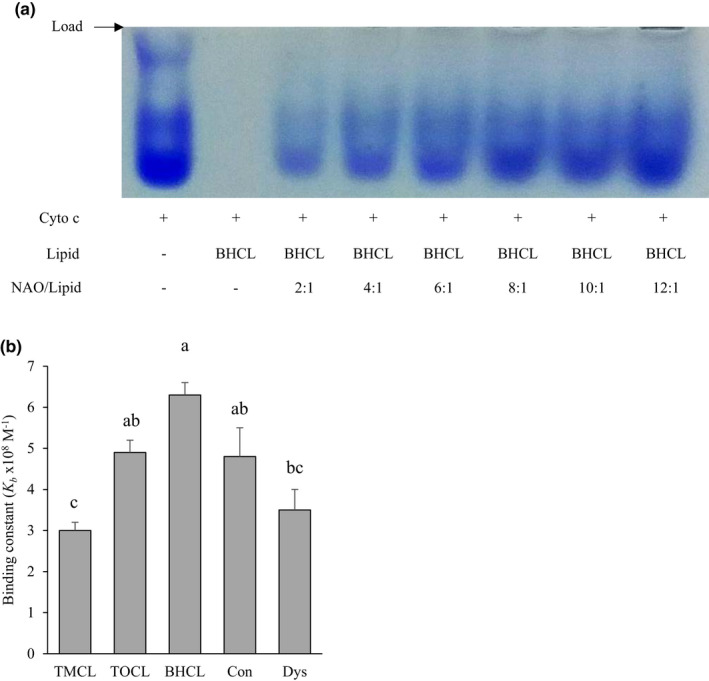
Binding affinity of cytochrome c is highest to tetralinoleoyl‐rich cardiolipin liposomes. (a) Native polyacrylamide gel electrophoresis of cytochrome c incubated with BHCL and varying concentrations of NAO and (b) resulting binding constants of the different cardiolipin liposomes. Values are mean ±SEM, n = 3 for each condition; values with the same letter are not significantly different from each other (*p* < 0.05); TMCL, 1’,3’‐bis[1,2‐dimyristoyl‐sn‐glycero‐3‐phospho]‐sn‐glycerol cardiolipin; TOCL, 1’,3’‐bis[1,2‐dioleoyl‐sn‐glycero‐3‐phospho]‐sn‐glycerol; BHCL, bovine heart cardiolipin; Con, liposome to broadly reflecting the percent cardiolipin composition of normal skeletal muscle mitochondria; Dys, liposome to broadly reflecting the percent cardiolipin composition of dystrophic skeletal muscle mitochondria; *K_b_*, binding constant x108 M^−1^ @ 90%; and NAO, 10‐N‐nonyl acridine orange.

The binding constant (*K_b_*) was dependent on cardiolipin acyl composition such that cytochrome c had the greatest affinity for BHCL and TOCL‐containing membranes compared to TMCL (Figure 4). When comparing cytochrome c's binding affinity for mixed CL membranes, cytochrome c binding affinity for Dys was significantly lower compared to BHCL (and similar to TMCL) but not statistically different from Con liposomes. *K_b_* did not significantly correlate with cytochrome c peroxidase activity (data not shown).

## 4 DISCUSSION

The present study investigated the relationship between liposomes containing CL species with differing acyl chain compositions and cytochrome c form and function as a peroxidase. Specifically, this is the first study to examine the effect of liposomes broadly reflecting the relative CL composition of both healthy and dystrophic muscle mitochondrial membranes, on cytochrome c conformation and function. The major findings of this study are as follows: (a) The cytochrome c peroxidase activity is increased in the presence of 50 µM H_2_O_2_ and liposomes containing unsaturated CL, provided CL species are non‐oxidized, (b) the presence of CL, regardless of acyl chain composition, influenced the folding kinetics of cytochrome c and when focused on Q band maximal absorbance, positively correlated with cytochrome c peroxidase activity, and (c) cytochrome c binding affinity to CL liposomes is side chain dependent such that binding affinity increased with the number of double bonds.

### 4.1 Cytochrome c form and function as a peroxidase are influenced by CL side chain composition

Our results suggest the importance of at least one double bond in cardiolipin‐containing liposomes during the transition of cytochrome c to a peroxidase, which is consistent with the previous literature (Abe et al., 2011; Belikova et al., 2006). The presence of CL with side chains that contain at least one double bond (BHCL or TOCL) resulted in greater binding (higher *K_b_* compared to TMCL) of cytochrome c to CL, which has also been reported previously (Belikova et al., 2006). However, to our knowledge, this is the first report of cytochrome c conformational change, specifically Q band peak absorbance, positively correlating with the peroxidase activity.

In its native form, cytochrome c is hexacoordinated with the fifth and sixth ligands provided by His18 and Met80, respectively (Elove et al., 1994). When cytochrome c acts as an electron shuttle, electrostatic forces loosely bind it to the IMM (Tuominen et al., 2002). The conformational change of cytochrome c into a peroxidase involves a tight interaction with the membrane (Li et al., 2019; Sinibaldi et al., 2017). Specifically, cytochrome c hydrophobically binds to CL, resulting in protein conformational changes (Tuominen et al., 2002). The hydrophobic binding requires one side chain of CL to extend into a cleft of cytochrome c created by the removal of cytochrome c's Met80 ligand (Alvarez‐Paggi et al., 2017; Rytomaa & Kinnunen, 1995; Tuominen et al., 2002). This extended lipid anchoring is what causes a conformational change in the heme environment (Mugnol et al., 2008), resulting in increased peroxidase activity (Kagan et al., 2009; Mohammadyani et al., 2018).

Conformational changes to cytochrome c's structure can be measured spectrophotometrically. The spectrophotometric changes, specifically increased Q band peak absorbance in cytochrome c interacting with CL containing liposomes compared to cytochrome c alone, suggest CL side chain‐specific modification to cytochrome c protein conformation (Droghetti & Smulevich, 2005; Mugnol et al., 2008; Tomaskova et al., 2010; Wiederkehr et al., 2009). Changes to the Q band have previously been reported in the literature, specifically Q band maximal absorbance decreased when cytochrome c is denatured (Mugnol et al., 2008; Tomaskova et al., ,^2010^, ^2018^). Thus, in the presence of CL‐containing liposomes with at least one double bond, there is transformation of cytochrome c to a peroxidase, preventing H_2_O_2_‐mediated damage to cytochrome c and resulting in peroxidase activity (Tomaskova et al., 2010). Greater conformational changes in cytochrome c were induced by BHCL compared to TOCL, but this did not translate to differences in peroxidase activity. Although cardiolipin peroxidation was not measured after incubation with cytochrome c, the previous literature has demonstrated that highly unsaturated fatty acids that contain multiple double bonds are more susceptible to lipid peroxidation (Hulbert et al., 2014). It is possible that cytochrome c preferentially peroxidized TLCL acyl chains and due to the lower degree of unsaturation, and TOCL acyl chains were less likely peroxidized. This may help explain the positive correlation, although indirectly related, between maximal Q band absorption and peroxidase activity.

### 4.2 Characteristics of cytochrome c peroxidase in the presence of mixed cardiolipin liposomes

Mitochondrial membranes are composed of different CL acyl chain compositions rather than a single species with homogeneous acyl chains. Dystrophic muscle contains proportionally more monounsaturated and less polyunsaturated phospholipid acyl side chains compared to healthy controls (Benabdellah et al., 2009; Tahallah et al., 2008; Touboul et al., 2004), a trend that extends to CL (Zibamanzarmofrad, 2015; Zibamanzarmofrad et al., 2013). Although not perfect and limited by commercially available CL species, liposomes containing 39% TLCL, 6% TOCL, and 5% TMCL or 28% TLCL, 17% TOCL, and 5% TMCL were used to broadly mimic the relative CL acyl chain composition of healthy and dystrophic skeletal muscle mitochondrial membranes, respectively.

**FIGURE 5:**
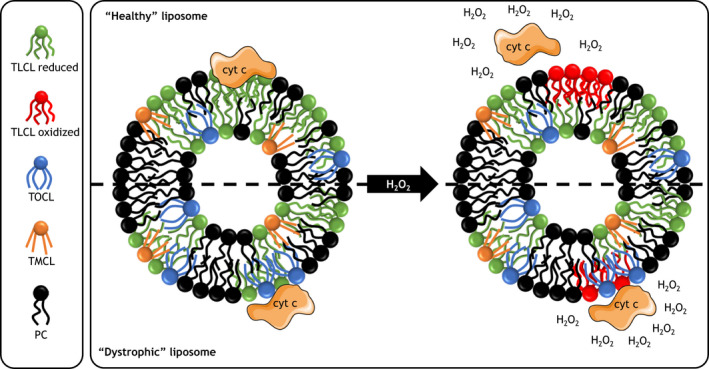
Proposed impact of increased monounsaturated and decreased polyunsaturated cardiolipin on cytochrome c form and function. In liposomes reflecting the relative cardiolipin composition of *healthy* muscle mitochondria, cytochrome c is tightly bound. In the presence of hydrogen peroxide, cytochrome c undergoes a transformation into a peroxidase, resulting in the oxidation of polyunsaturated cardiolipin and release of cytochrome c. In liposomes reflecting the relative cardiolipin composition of *dystrophic* muscle mitochondria, specifically more monounsaturated and less polyunsaturated, cytochrome c is more loosely bound due to a lower binding constant compared to *healthy* liposomes. In the presence of hydrogen peroxide, cytochrome c transforms into a peroxidase but may not oxidize the less susceptible monounsaturated CL and may remain associated with the liposome; TMCL, tetramyristoyl cardiolipin; TOCL, tetraoleoyl cardiolipin; TLCL, tetralinoleoyl cardiolipin; PC, phosphatidylcholine; cyt c, cytochrome c; and H_2_O_2_, hydrogen peroxide.

Considering the findings when examining the influence of liposomes composed of CL with one species of acyl chain on cytochrome c form and function, mixed liposomes follow a similar pattern. Specifically, the presence of CL acyl chains with at least one double bond led to the transformation of cytochrome c into a peroxidase due to conformational changes in the heme environment, similar to BHCL and TOCL. Alternatively, some outcome measures were intermediate to BHCL and TMCL, such as the binding coefficient. As a result, we have suggested a working hypothesis in which a shift in CL acyl chain composition seen with muscular dystrophy may influence the interaction between cytochrome c and CL (Figure 5).

### 4.3 Future directions

In healthy muscle mitochondria under normal conditions, cytochrome c contributes to oxidative phosphorylation. The limited amount of CL on the IMS‐facing leaflet of the IMM (Horvath & Daum, 2013) promotes the loose electrostatically bound cytochrome c to shuttle electrons between complexes III and IV (Huttemann et al., 2011). With an apoptotic trigger, TLCL on the matrix‐facing leaflet of the IMM translocates to the IMS‐facing leaflet (Kagan et al., 2015) and hydrophobically binds with cytochrome c, transforming it into a peroxidase (Kagan et al., 2009; Mohammadyani et al., 2018). Cytochrome c peroxidase catalyzes the homolytic cleavage of H_2_O_2_, resulting in the formation of water and oxidization of neighbouring TLCL, an event that precedes the release of cytochrome c (Gonzalvez & Gottlieb, 2007; Kagan et al., 2005). As evident by our findings (Figure 2a and 2b), oxidized BHCL (which is ≥80% TLCL) does not promote the cytochrome c peroxidase activity, which may be due to the lack of interaction between oxidized TLCL and cytochrome c (Kagan et al., 2005). Future research should examine the extent of cytochrome c binding to mitochondrial membranes with differing CL saturations and with differing CL concentrations (i.e., healthy vs apoptotic environment) and different oxidation levels to determine if the findings hold true in vivo. However, a limitation is using liposomes with physiological levels of CL [10–15% found in skeletal muscle mitochondria (Stefanyk et al., 2010)] to examine their influence on cytochrome c form and function in vitro. Previous research has demonstrated that low CL concentrations (20%) impair the binding of cytochrome c to the liposome (Schweitzer‐Stenner, 2018).

In contrast, dystrophic skeletal muscle mitochondria have a decreased polyunsaturated and increased monounsaturated CL, potentially influencing the interaction between cytochrome c and the IMM. After an apoptotic trigger and the translocation of charged but not neutral phospholipids, including CL, by mitochondrial phospholipid scramblase‐3 to the IMS‐facing leaflet of the IMM (Dudek, 2017), cytochrome c becomes a peroxidase. However, despite having a potentially lower binding affinity to cytochrome c, due to the lower oxidation potential of monounsaturated compared to polyunsaturated acyl side chains (Hulbert et al., 2014), cytochrome c is less likely to be released and remain hydrophobically attached. Future research should determine how lower binding affinity and increased peroxidase activity may be related in vivo to mitochondrial membrane composition and apoptosis.

## 5 CONCLUSION

This was the first study to describe the effect of BHCL, TOCL, and TMCL‐based liposomes on the conformation, function, and binding affinity of cytochrome c, in addition to identifying the relationship between these properties as they relate to the transition from cytochrome c to a peroxidase. It was also the first to determine the effects of liposomes mimicking the membranes of healthy and dystrophic mitochondria on these properties. Our findings suggest a positive correlation between CL unsaturation and cytochrome c's binding affinity to the liposome. Cytochrome c's binding to the liposome contributes to conformational and functional changes that promote peroxidase activity.

## CONFLICT OF INTEREST

The authors have none to declare.

## AUTHORS CONTRIBUTIONS

JAW performed the experiments, analyzed the data, and wrote the manuscript. SS assisted in working up the peroxidase activity assay and edited the manuscript. PJL developed the overall study, analyzed the data, and wrote and edited the manuscript.
